# Large scale dataset of real space electronic charge density of cubic inorganic materials from density functional theory (DFT) calculations

**DOI:** 10.1038/s41597-022-01158-z

**Published:** 2022-02-21

**Authors:** Fancy Qian Wang, Kamal Choudhary, Yu Liu, Jianjun Hu, Ming Hu

**Affiliations:** 1 State Key Laboratory of High-end Server & Storage Technology, Inspur Electronic Information Industry Co., Ltd, Beijing, 100085 China; 2 Materials Science and Engineering Division, National Institute of Standards and Technology, Gaithersburg, MD 20899 USA; 3 Theiss Research, La Jolla, CA 92037 USA; 4 Department of Computer Science and Engineering, University of South Carolina, Columbia, 29208 South Carolina United States; 5 Department of Mechanical Engineering, University of South Carolina, Columbia, 29208 South Carolina United States

**Keywords:** Electronic structure, Electronic properties and materials

## Abstract

Driven by the big data science, material informatics has attracted enormous research interests recently along with many recognized achievements. To acquire knowledge of materials by previous experience, both feature descriptors and databases are essential for training machine learning (ML) models with high accuracy. In this regard, the electronic charge density *ρ*(*r*), which in principle determines the properties of materials at their ground state, can be considered as one of the most appropriate descriptors. However, the systematic electronic charge density *ρ*(*r*) database of inorganic materials is still in its infancy due to the difficulties in collecting raw data in experiment and the expensive first-principles based computational cost in theory. Herein, a real space electronic charge density *ρ*(*r*) database of 17,418 cubic inorganic materials is constructed by performing high-throughput density functional theory calculations. The displayed *ρ*(*r*) patterns show good agreements with those reported in previous studies, which validates our computations. Further statistical analysis reveals that it possesses abundant and diverse data, which could accelerate *ρ*(*r*) related machine learning studies. Moreover, the electronic charge density database will also assists chemical bonding identifications and promotes new crystal discovery in experiments.

## Abstract (summary)

**Table:** 

Measurement(s)	electronic charge density
Technology Type(s)	computational methods
Factor Type(s)	inorganic material

Machine-accessible metadata file describing the reported data: 10.6084/m9.figshare.17694158

## Background & Summary

The electronic charge density (ECD) in real space, denoted as *ρ*(*r*), is a basic yet informative observable quantity of materials in physics. Dated back to 1964, density functional theory (DFT) was discovered by Hohenberg and Kohn, who proved that the properties of materials at their ground state can be entirely and exclusively determined by *ρ*(*r*)^1,2^. This theorem has been widely used and applied to multiple physical systems from microscale molecules to macroscale crystals, significantly accelerating our understanding and manipulation of the nature of the world. For example, the bonding characters between neighbouring atoms, a very fundamental yet complex concept (covalent, ionic, metallic bonds), can be fully described by ECD, rather than the simple bonding chemistry. With this property, novel materials with specific structures and target properties^3,4^ could be artificially designed, which is one of the most crucial issues in modern crystallography and inverse-materials design^3^. In addition, most electronic, magnetic, optical properties and their couplings, such as electrostatic moments, potentials and interaction energies, spin susceptibility, light absorption, and electromagnetic responses, etc, could be directly obtained starting from the ECD *ρ*(*r*) according to the modern band structure theory^2^. Because of these advantages, the basic quantity *ρ*(*r*) possesses broad applications, such as identifying the binding sites in host-guest compounds^5^, computing infrared intensities^6,7^, revealing structural stability, simulating scanning tunnelling microscopy images^8^ and so on^9–11^. For instance, Shen *et al*. have recently reported a charge-density-based general cation insertion algorithm for generating and designing new Li-ion cathode materials^11^, and Hu *et al*. have made efforts to predict mechanical elastic properties of materials from their *ρ*(*r*)^10^.

From the experimental perspective, the ECD *ρ*(r) can be probed by high-resolution electron diffraction^4,12–14^ and transmission electron microscopy, and then subjected to Bader’s Quantum Theory of Atoms in Molecules (QTAIM)^15,16^ analysis for extracting bonding information based on multipole models (MM) density^14,17^. However, the flaw of such technique resides in collecting raw and accurate metadata of inorganic materials, especially for those high-symmetric dense solids with extended structures and heavy atoms. This is because, in such materials, the scattering from the valence electrons is minute compared to that from core electrons. Consequently, systematic errors such as extinction and absorption are critical^17^. Accordingly, unlike the widely explored molecular systems, very few ECD *ρ*(*r*) of inorganic materials were acquired even using the modern techniques. On the contrary, from the theoretical point of view, the ECD *ρ*(*r*) of inorganic materials can be accurately simulated by using *ab initio* calculations according to the density functional theory (DFT). Even though quantum chemistry approaches are more immune to artificial parameters, the computational demanding of DFT is more appropriate for realistic materials, rather than small molecules. Hence, the DFT method with sufficient predictive ability is powerful in dealing with ECD *ρ*(*r*) of inorganic materials, and thus giving better assistance to experiments.

In the modern science and technology world, big data-driven science has become the fourth science paradigm^18,19^, thanks to the significantly increased computing power and the huge data generated every day. Utilizing the machine learning (ML) algorithms to solve problems in material science has significantly boosted the development of material informatics^19–23^, among which bypassing the DFT calculations to directly predict the fundamental *ρ*(*r*) of materials has attracted immense attentions^24–27^. Nevertheless, most of the recent progresses were confined to the molecular systems^28,29^, mainly because the systematic ECD *ρ*(*r*) database of inorganic materials is still in its infancy.

**Fig. 1:**
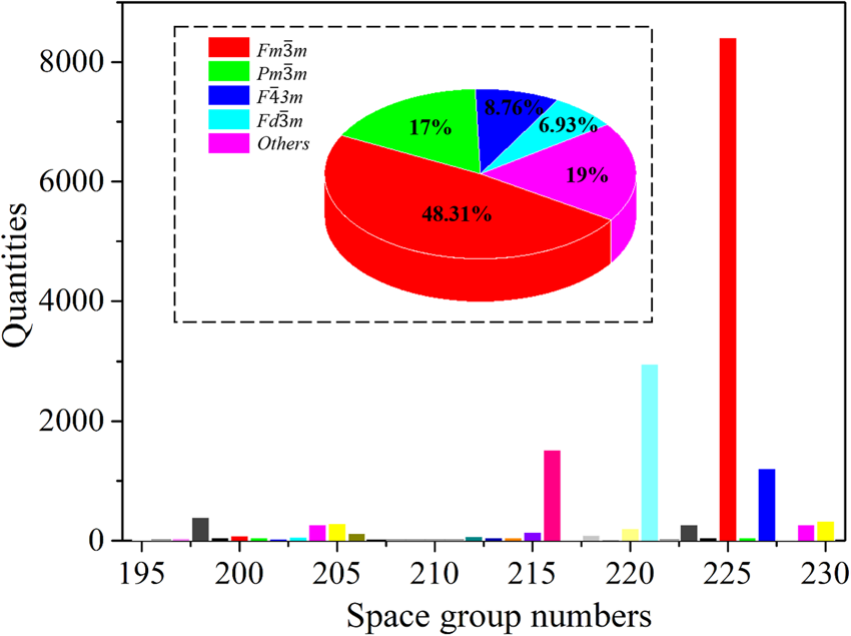
The number of materials with respect to each space group in ECD-cubic database. The insertion is the corresponding percentage distribution.

**Fig. 2:**
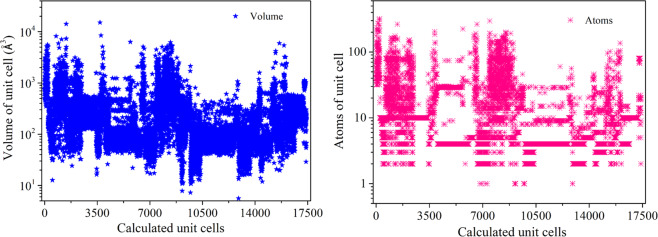
(**a**) The distributions of volume and (**b**) atom numbers in a unit cell for all the materials in ECD-cubic database.

**Fig. 3:**
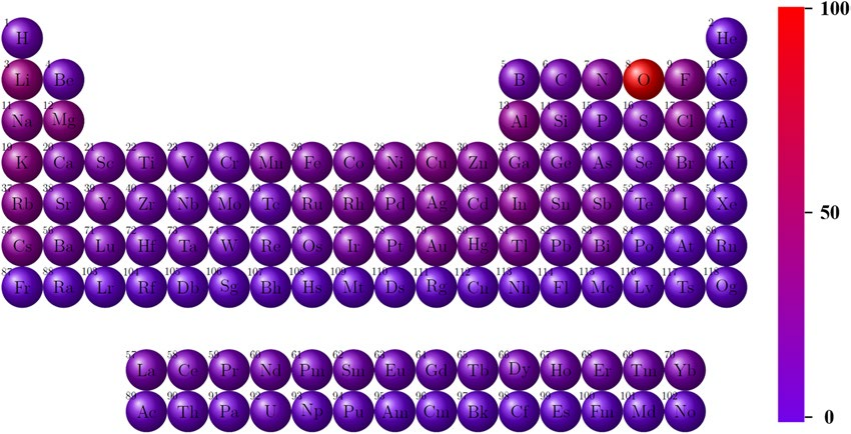
The distribution of elements for all the materials in ECD-cubic database.

Based on the above discussions, in this study, we construct the ECD-cubic, an ECD *ρ*(*r*) database of inorganic materials by using the state-of-the-art first-principles calculations. Because of the expensive yet limited computational resources, we focus on materials in cubic symmetry where the *ρ*(r) are more efficient to calculate (in computation time per atom). The *ρ*(r) of materials with other symmetries, such as hexagonal, orthorhombic and monoclinic, will be successively included in our future work. So far, the ECD-cubic database contains the *ρ*(*r*) of 17,418 materials, which are all selected from the Materials Project database^30,31^ and possess cubic structures. The distributions of space group, volume and number of atoms in the unit cell and elements of the ECD-cubic database are statistically analysed and the results are given in Figs. 1–3. We can see that the space groups of the materials in the ECD-cubic database span from *P23* (195) to $la\bar{3}d$ (230), where the proportions of the top three, namely the $Fm\bar{3}m$ (225), $Pm\bar{3}m$ (221), $F\bar{4}3m$ (216) are 48.31%, 17% and 8.76%, respectively. It is found that the volumes of the unit cells in ECD-cubic database span five orders of magnitude, from the smallest one with 5.6Å^3^ (mp-998866) to the largest one with 15,120Å^3^ (mp-1172909), and most of them are in the range of 30–1,000Å^3^ (~93% in the database). Consistently, the number of atoms in the unit cell also vary in a wide range. For instance, the Rb_3_Sc_2_(AsO_4_)_3_ (mp-1205185) and Te(CF_2_)_4_ (mp-1204577) have extraordinarily complicated atomic geometry with 320 and 312 atoms in the unit cell, respectively, while a lot of metals with the face-centered cubic (FCC) structure (mp-81, mp-10740, mp-20483, mp-612118, etc) only have one atom in their primitive cells. The colour bar displayed in Fig. 3. distinguishes the percentage of each element involved in materials, where the red and purple indicate higher and lower ratios, respectively. We can see that the ECD-cubic database consists of 89 kinds of elements, where the O element (6.62%) far exceeds others, and the alkali metals, namely, the Li (2.72%), K (2.34%) and Mg (2.33%) elements afterwards.

All the features above demonstrate that the constructed ECD-cubic database is formed by high-quality, abundant, and diverse data, which is an essential prerequisite for ECD related ML studies in material informatics. Simultaneously, it will also provides good opportunities to crystal engineering and gives better guidance for the chemical bonding identifications in experiments.

## Methods

In this section, we give a brief introduction to the methods employed in this study, including the general theory of ECD *ρ*(*r*) adopted by VASP (Vienna ab-initio simulation package) software, the workflow that we select materials, and all the parameters used in the DFT calculations.

### General theory

In DFT, the relationship between ECD *ρ*(*r*) and wave function $\Psi \left(r,{r}_{2},\cdots ,{r}_{N}\right)$ is given by:

$$\rho \left(r\right)=N\int {d}^{3}{r}_{2}\cdots \int {d}^{3}{r}_{N}\Psi * \left(r,{r}_{2},\cdots {r}_{N}\right)\Psi \left(r,{r}_{2},\cdots {r}_{N}\right)$$

The total number of electrons (*N*) in a unit cell are equal to the integral of ECD *ρ*(*r*) over the entire volume (*V*_*un*_). After discretization, the relationship could be expressed as:

$$N={\int }_{V}\rho \left(r\right)dV={\sum }_{i=1}^{NGXF\ast NGYF\ast NGZF}\rho \left({r}_{i}\right)\frac{{V}_{un}}{NGXF\ast NGYF\ast NGZF}$$

where the *NG*(*X*, *Y*, *Z*)*F* are the fine Fast Fourier Transform (FFT) grids in the reciprocal space along the *x*, *y* and *z* directions, respectively. A series of discrete values of $\rho \left({r}_{i}\right){V}_{un}$ at each fine FFT-grid are recorded in the CHG file, carrying all the desired information of *ρ*(*r*). In non-spin polarized calculations for non-magnetic materials, the CHG only contains total electronic charge density $\left(\rho \left(r\right)=\rho {\left(r\right)}_{spinup}+\rho {\left(r\right)}_{spindown}\right)$, while for those spin-polarized calculations for magnetic materials, a spin electronic charge density $\left(\rho \left(r\right)=\rho {\left(r\right)}_{spinup}-\rho {\left(r\right)}_{spindown}\right)$ will be additionally given.

### Workflow

All materials are downloaded from the Material Project database^30,31^, which is one of the widely used databases in material informatics since it contains more than 144,595 inorganic compounds with three-dimensional structural information. On account of the extremely expensive computational cost of the DFT calculations, the cubic symmetry is treated as the criterion when we select materials, leaving 18,494 candidates. Out of these candidates, we have calculated the electronic charge density *ρ*(*r*) of 17,418 materials after filtering out the structures with incomplete information or the calculations cannot achieve good self-consistent field convergence after several tries. The ECD *ρ*(*r*) of the remaining materials with non-cubic symmetries would be available in the future.

### Density functional theory calculations

All the first-principles calculations are carried out using the projector augmented wave (PAW) method^32,33^ as implemented in the Vienna Ab initio Simulation Package (VASP) based on the density functional theory (DFT). We start by optimizing each crystal structure, where both the atomic positions and lattice constants are fully allowed to relax in spin-unrestricted mode and without any symmetry constraints. These calculations are performed until the maximal Hellmann-Feynman force component smaller than 10^−3^ eVÅ^−1^, and the total energy convergence tolerance is set to be 10^−6^ eV. To obtain accurate lattice parameters^34–36^, the Opt-B88vdW functional^37^ is taken into account to deal with the long term interactions in the exchange-correlation interaction. The k-point grids (KPOINTS) for each material used to calculate their CHGs are the same KPOINTS files as used in static calculations in Materials Project database (downloaded in around June 2020), which have been proved to achieve good convergence in previous works. All the required KPOINTS files are obtained by using the Pymatgen (Python Materials Genomics), which is a robust, open-source python library for materials analysis. The choice of kinetic energy cut-off of planewave functions for each material is also according to the Materials Project database. After fully converged, the ECD *ρ*(*r*) of all the materials are calculated separately with energy convergence threshold is set to be 10^−6^ eV.

## Data Records

As mentioned above, the ECD-cubic database is formed by the *ρ*(*r*) of 17,418 inorganic materials along with their atomic structures. To be consistent with the Material Project database, we continue to use the same material ID for identifying them. The metadata of each material is stored in the Javascript Object Notation Files (JSON) format, denoted as mp-id.json, which can be easily integrated with other databases such as MongoDB. All the entries including the keys and their corresponding descriptions of the JSON file are listed in Table 1. Moreover, we also provide a python script to parse the JSON file to the standard CHG format for visualizing or restarting the VASP calculations. According to the previous study, the quality of the data is determined by its coverage of the chemical-property space of interest as well as the uncertainty associated with the data^38^. In the ECD-database, all the ρ(r) are created by consistent DFT calculations, thus largely removing its uncertainty. While for separating the chemical-property space of interest, herein, we provide a structural list recording the IDs with the corresponding energy above hull of each material. Such a list and the ECD-database are made available through Figshare repository^39^. The same copy is also uploaded to our Carolina Materials Database (http://www.carolinamatdb.org/). Besides, to enhance the reproducibility of this work, all the raw input files, namely the POSCAR, KPOINTS and INCAR for calculating the electronic charge density of each material can be acquired via Carolina Materials Database.

**Table 1:** The keys and their corresponding descriptions of the JSON file for each material.

Keys	Description
system	The name of calculated material, the same as the content in the 1^st^ line of CHG file.
vector	Vector, usually 1.0, the same as the content in the 2^nd^ line of CHG file.
lattice	Lattice constants along the *x*, *y* and *z* directions, respectively, the same as the content from 3^rd^ - 4^th^ line of CHG file.
elements	The elements involved in materials, the same as the content in the 5^th^ line of CHG file.
elements_number	The quantities of each element listed above, the same as the content in the 6^th^ line of CHG file.
coor_type	The type, usually direct, the same as the content in the 7^th^ line of CHG file.
coordinates	The atomic coordinates along *x*, *y* and *z* directions in materials, the same as the content in the 8^th^-(8 + 3 *N*)^th^ line of CHG file, where *N* is the number of atoms in the cell.
FFT	The FFT grids (*NGXF*, *NGYF* and *NGZF*) used in calculations along the *x*, *y* and *z* directions, the same as the content after coordinates in CHG file.
charge	The calculated electronic charge density components based on the FFT grids. Note: if the materials without magnetism, such entry only contain the total charge density, the number of components equal to NGXF*NGYF*NGZF; while for materials with magnetism, such entry additionally contain the spin charge density behind the total charge density, the number of components equal to 2*NGXF*NGYF *NGZF+*1*

We would like to give some comparison between our calculations and other publicly available datasets. The Material Project database includes several datasets such as band structures, piezoelectric tensors, and elastic properties, yet totally excludes the electronic charge densities. Although a few CHGCARs of materials could be found in the NOMAD repository^40^, there are some uncertainties in terms of the quality of those CHGCARs. First, the CHGCARs stored in NOMAD are generated in the calculations of structure optimization, not the self-consistent process. Such CHGCARs are usually used to restart VASP calculations, not for the electronic charge densities analysis. Second, the parameters used for calculating the CHGCARs are missing, thus users may not perform reliable subsequent processing from these data. Besides, we extract initial structures of each material from the Material Project database and re-optimize them using the opt-B88vdW functional instead of the GGA/PBE functional used in the Material Project database. This would be more accurate because the opt-B88vdW functional has been proved to give much improved lattice parameters for both van der Waals (vdW) and non-vdW solids^34^, which are essential to the calculations of accurate electronic charge density. In addition, such functional is usually adopted in the construction of many other properties related database^8,34,36,41^, hence our datasets would be useful for further analysis and comparisons since consistent computational procedures are adopted.

## Technical Validation

**Fig. 4:**
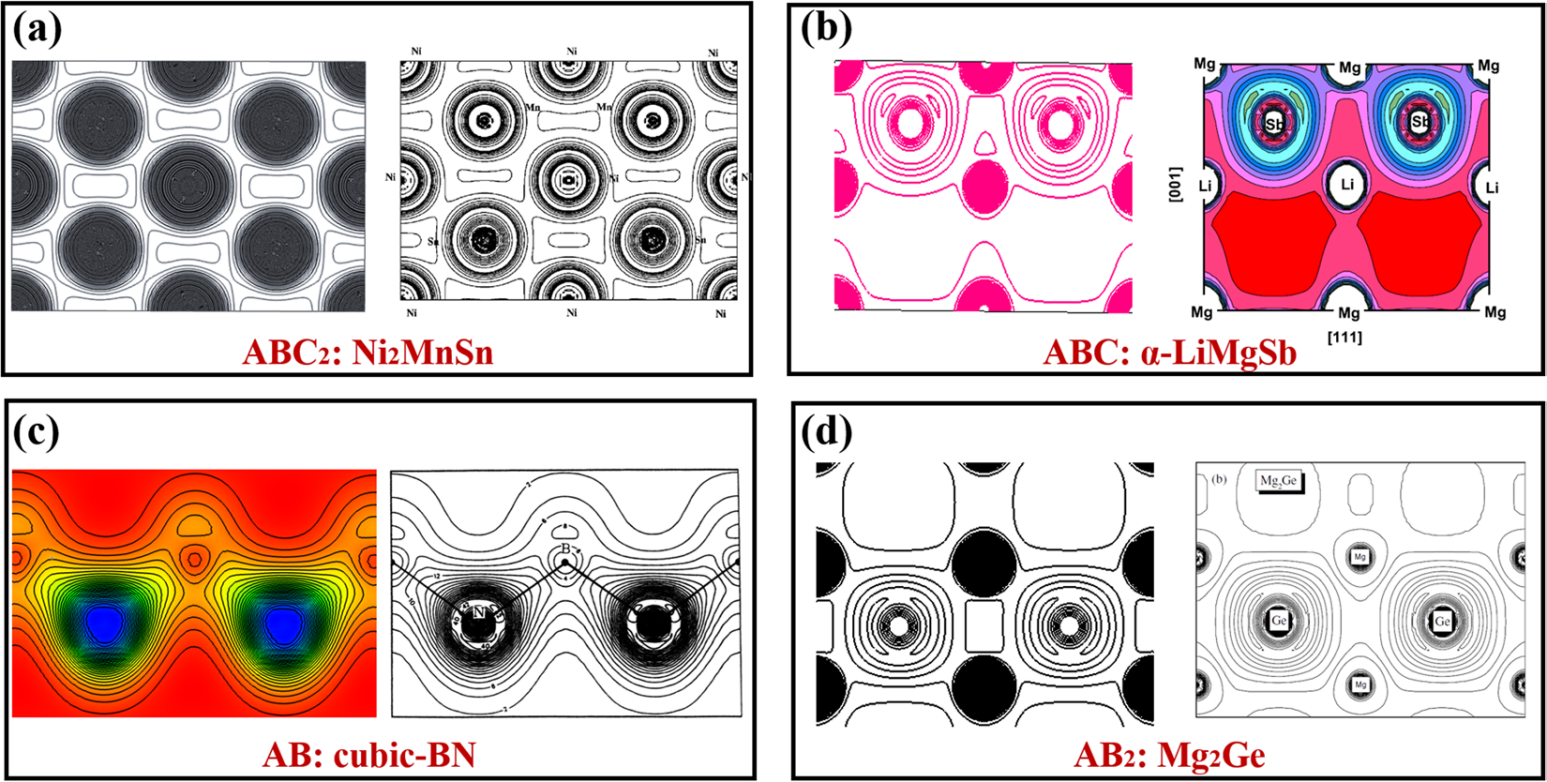
The calculated patterns of electronic charge density *ρ*(*r*) of (**a**) ABC_2_ type, Ni_2_MnSn [The right panel reprint with permission from ref. ^51^. Copyright 2001 American Physical Society], (**b**) ABC type, *α*-LiMnSb [The right panel reprint with permission from ref. ^47^. Copyright 2010 Elsevier], (**c**) AB type, cubic-BN [The right panel reprint with permission from ref. ^52^. Copyright 1986 American Physical Society] and (**d**) AB_2_ type, Mg_2_Ge [The right panel reprint with permission from ref. ^48^. Copyright 2005 Wiley], respectively.

**Fig. 5:**
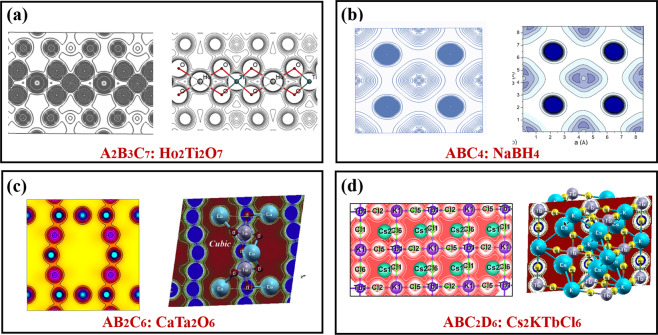
The calculated patterns of electronic charge density *ρ*(*r*) of (**a**) A_2_B_3_C_7_ type, Ho_2_Ti_2_O_7_ [The right panel reprint with permission from ref. ^53^. Copyright 2017 Elsevier], (**b**) ABC_4_ type, NaBH_4_ [The right panel reprint with permission from ref. ^50^. Copyright 2010 American Physical Society], (**c**) AB_2_C_6_ type, CaTa_2_O_6_ [The right panel reprint with permission from ref. ^42^. Copyright 2019 Elsevier] and (**d**) ABC_2_D_6_ type, Cs_2_KTbCl_6_ [The right panel reprint with permission from ref. ^47^. Copyright 2010 Elsevier], respectively.

**Fig. 6:**
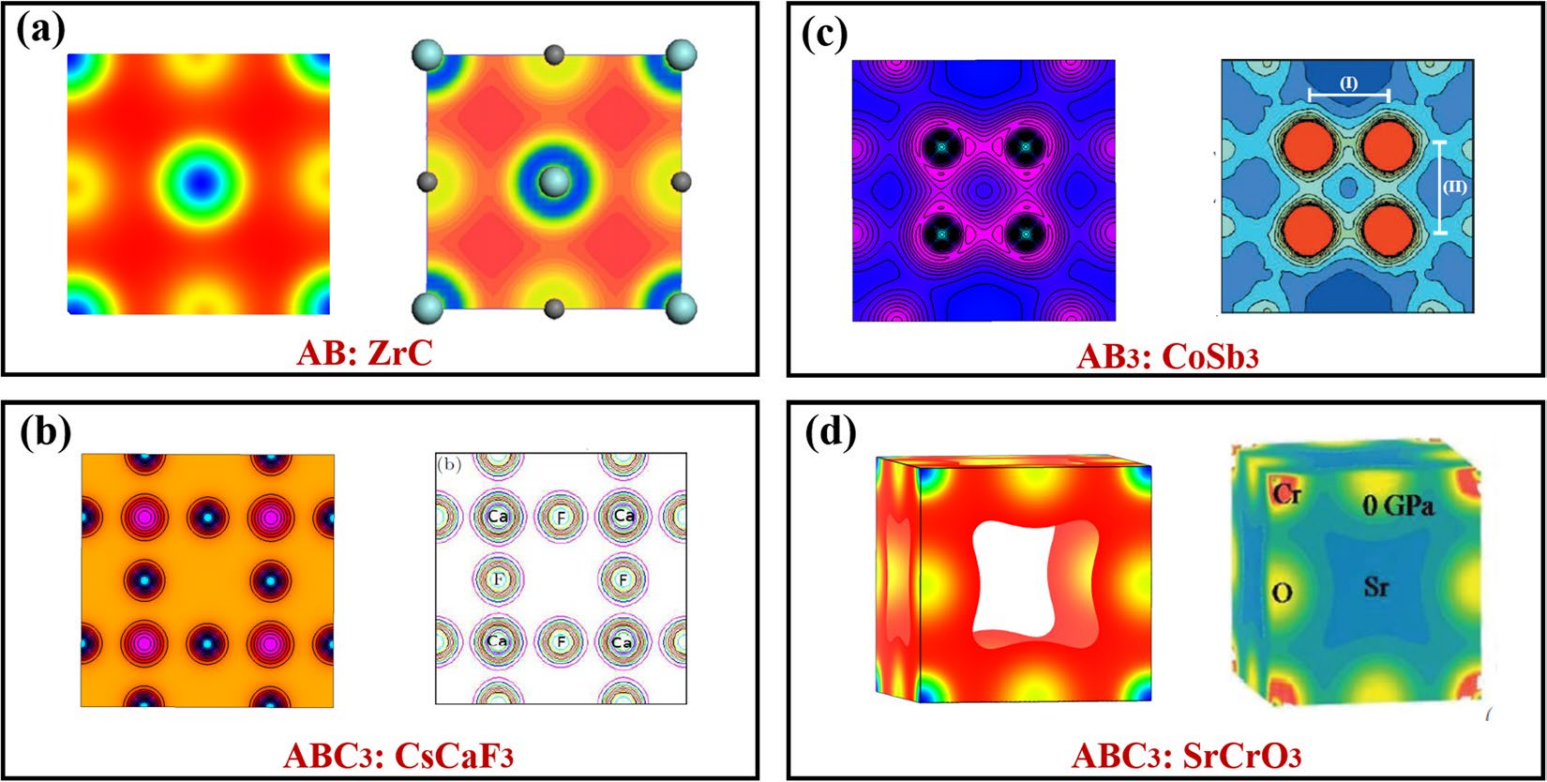
The calculated patterns of electronic charge density *ρ*(*r*) of (**a**) AB type, ZrC, [The right panel reprint with permission from ref. ^46^. Copyright 2011 Elsevier], (**b**) AB_3_ type, CoSb_3_ [The right panel reprint with permission from ref. ^55^. Copyright 2007 American Physical Society] and (**c**,**d**) ABC_3_ type, CsCaF_3_ [The right panel reprint with permission from ref. ^43^. Copyright 2012 IOP Science], and SrCrO_3_[The right panel reprint with permission from ref. ^54^. Copyright 2020 IOP Science], respectively.

**Fig. 7:**
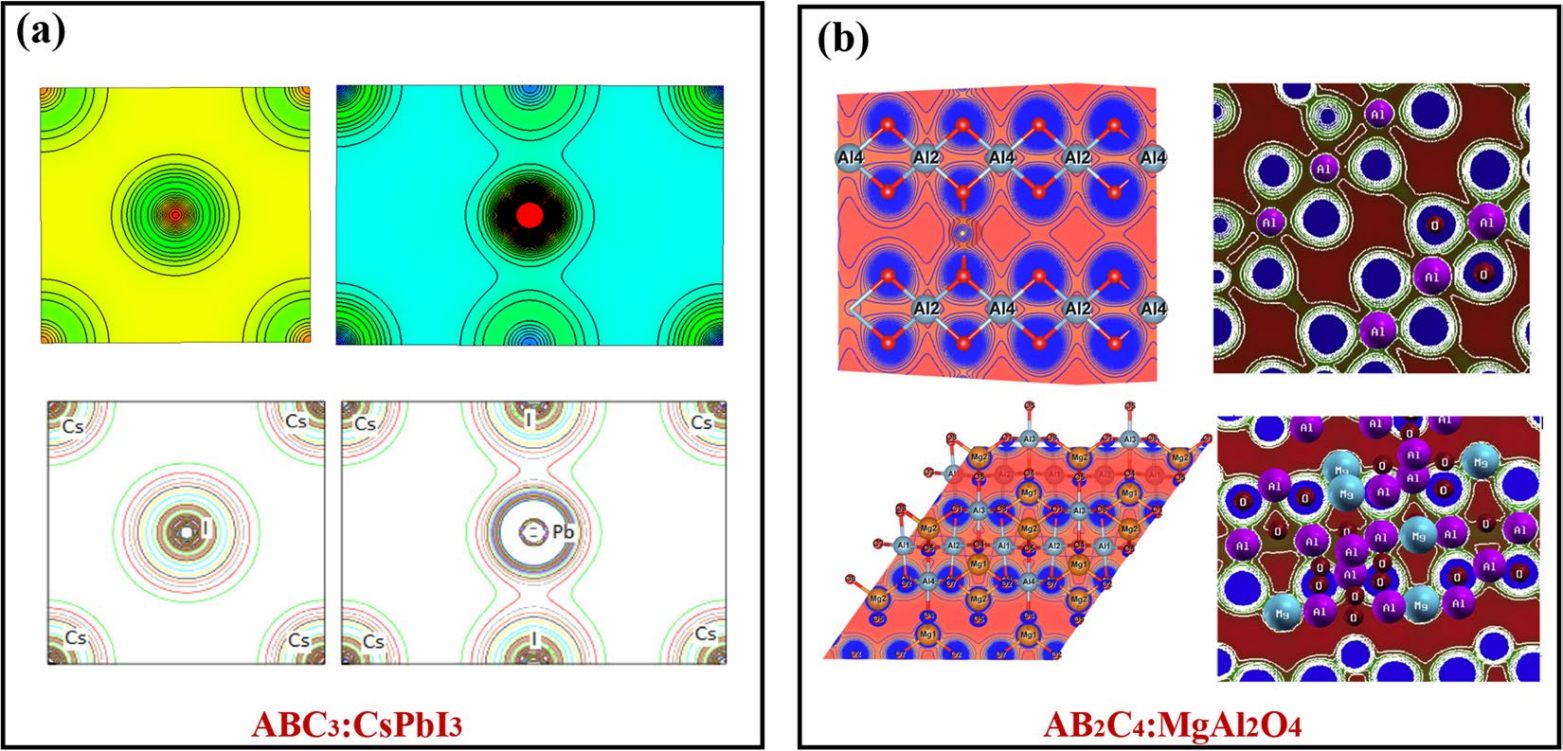
The calculated patterns of electronic charge density *ρ*(*r*) of (**a**) ABC_3_ type, CsPbI_3_ [The right panel reprint with permission from ref. ^44^. Copyright 2011 Elsevier] and (**b**) AB_2_C_4_ type, MgAl_2_O_4_ [The right panel reprint with permission from ref. ^49^. Copyright 2014 Elsevier].

The calculated electronic charge density *ρ*(*r*) is a widely accepted quantity for predicting physical properties^34^ benefitting from the significant predictive power of the state-of-the-art DFT calculations. Here we elaborate several patterns of the calculated *ρ*(*r*) and compare them with those reported in previous theoretical or experimental studies, to further verify the correctness of our simulations. Since there are over 17,418 electron density files calculated in our work, and it is unlikely to visualize all of them in the manuscript. Instead, we only show a few visualizations from scratch, without any biased selections. Hence, a screening process is essential for choosing the representative materials for visualizing. We start with figuring out the simplest chemistry formulae of all materials in the ECD-cubic database, to specify the differences among materials. The results show that there are 507 types of formulae with the simplest chemistry in the ECD-cubic database, where the proportions of the formulae ABC_2_, ABC_2_D_6_ and AB_3_ ranking top three with the value of 24.92%, 12.11% and 9.86%, respectively. Besides, the total proportions of the top 13 types account for 77% and each of them contributes more than 1%, thus screened as our target materials. Ultimately, fourteen materials with well-characterized experimental or computational images in the literature, are chosen and their corresponding electronic charge density *ρ*(*r*)^42–54^ are shown in Figs. 4–7. To further clarify, the patterns of each material are framed in a black rectangle, where the left panel shows our simulated pattern while the right panel is the reference reprinted from previous studies. Notably, the space group, elements, the size of unit cells or structures of all the selected materials are not restricted, thus making the technical validation reliable.

The selected materials with diverse crystal structures and elements, cover extensive research areas, such as the full Heusler alloys Ni_2_MnSn (mp-20440)^51^, the host-guest thermoelectric materials CoSb_3_^54,55^ (mp-1317), the alkaline tetrahydroborides NaBH_4_ (mp-976181)^50^, the spinel oxides MgAl_2_O_4_ (mp-3536)^49^, even the rare earth pyrochlores Ho_2_Ti_2_O_7_ (mp-33948)^53^, etc. Each material stands for one type, which has similar structures yet different chemical constituents. For example, the ABC type^47^ mainly consists of Nowotny-Juza A^I^B^II^C^V^, where A^I^ = Li, Na, Cu, Ag; B^II^ = Be, Mg, Zn, Cd; C^V = ^N, P, As, Sb, Bi. Meanwhile, the typical materials of ABC_3_ and AB_2_ type are cubic perovskites as well as IIA–IV antifluorite compounds^48^, respectively. This clearly proves that the materials we screened out could give comprehensive descriptions about the whole database, even if the limited number of *ρ*(*r*) of materials are visualized. We observe that all the electronic charge density *ρ*(*r*) patterns based on our calculations are in good agreement with those in the previous studies, confirming the technical validation of our results.

Next, several sources of inaccuracy during the dataset construction need to be discussed. The first limitation is that the magnetic orders of each case are not included in our massive calculations, mainly due to the absence of experimental data. In fact, the electron spin whether in ferromagnetic (FM) or antiferromagnetic (AFM) configurations, almost has no influence on the total electronic charge density ($\rho ={\rho }_{spinup}+{\rho }_{spindown}$) concerned there, yet slightly affects the spin electronic charge density (${\rho }_{spin}={\rho }_{spinup}-{\rho }_{spindown}$) in some cases. The reason is that the electron bonding (or antibonding) energy scale is on the order of a few electron volt (eV), while the spin polarization energy scale is usually a few tens of millielectron volt (meV). For example, except for considering the fine structure near the nuclei, the spin electronic charge density of CaMnO_3_^56^ shows much similarities between its AFM and FM phases, which is akin to that of other cubic perovskites, namely the KMnF_3_^57,58^, KNiF_3_^59^, KCuF_3_^60^, etc.

The second drawback is that the DFT calculation is conducted at absolute zero temperature (0 K), entirely ignoring the temperature effects on both electron and ion subsystems. This may induces some inconsistency between the theoretical and experimental results, especially when the latter is performed under high temperature. For instance, the covalent bond strength of NiO is increased with the sintering temperature, which can be clearly seen through its electronic charge density mapping^61^. The lattice constants of many materials would increase a bit from 0 K to finite temperature, which usually results in slight change in the spatial distribution of electronic charge density.

Third, some materials would undergo phase transition from 0 K to finite temperature. A typical example is that some cubic halide perovskites in ABC_3_ formula, where the B-sites ions possess *s*^*2*^ long pair electrons, such as CsPbX_3_ and CsSnX_3_, exhibit dynamic off-centering effect^62,63^. The ion position fluctuates between eight energetically favourable asymmetric configurations. Hence, the B site ions in these cubic systems is not located at the center of an octahedral coordination environment^64^. Such phenomenon is enhanced with the reduced temperature, which is beyond the discussion in this study.

Finally, the U parameters may induce some discrepancy of ρ(r) for strong correlation materials, such as transition metal oxides. However, we did not inherit the U parameters in this study due to the following reasons. First, only a few U parameters are provided in the Materials Project for transition metal oxides. This will make the calculations inconsistent if we only add the U parameters for some materials. Second, the U value is an empirical parameter in practice, so its value can be quite different across different studies or databases^65^. We would like to emphasize that there is no universal U values for any material that can well reproduce all physical properties, including lattice constants, elastic coefficients, band dispersion, phonon spectrum, etc. Hence, the choice of the U values may be determined case-by-case, which is beyond the scope discussed here.

## Data Availability

Python-language-based codes for converting the JSON file to the original CHG file generated by VASP of each case are provided, which can be downloaded through Figshare^39^ and our Carolina Materials Database (http://www.carolinamatdb.org/).
